# A pandemic *Vibrio parahaemolyticus* O3:K6 clone causing most associated diarrhea cases in the Pacific Northwest coast of Mexico

**DOI:** 10.3389/fmicb.2015.00221

**Published:** 2015-03-24

**Authors:** Lucio de Jesús Hernández-Díaz, Nidia Leon-Sicairos, Jorge Velazquez-Roman, Héctor Flores-Villaseñor, Alma M. Guadron-Llanos, J. Javier Martinez-Garcia, Jorge E. Vidal, Adrián Canizalez-Roman

**Affiliations:** ^1^Regional Doctorate Program in Biotechnology, School of Biological Chemical Sciences, Autonomous University of SinaloaCuliacán, Mexico; ^2^School of Medicine, Autonomous University of SinaloaCuliacán, Mexico; ^3^Pediatric Hospital of SinaloaCuliacán, Mexico; ^4^Rollins School of Public Health, Emory UniversityAtlanta, GA, USA; ^5^The Sinaloa State Public Health Laboratory, Secretariat of HealthCuliacán, Mexico

**Keywords:** serologic, isolation, *Vibrio parahaemolyticus*, biosurveillance, public health

## Abstract

Between September and October of 2004, more than 1230 cases of gastroenteritis due to pandemic O3:K6 strains of *Vibrio parahaemolyticus* (*V. parahaemolyticus*) were reported in the relatively small geographical area of Southern Sinaloa, a state located in Northwest Mexico. Since then, *V. parahaemolyticus*-associated gastroenteritis cases have gradually increased in prevalence spreading from south to north. The present study conducted an epidemiological surveillance of *V. parahaemolyticus* strains in both environmental and clinical samples along the Pacific coast of Sinaloa from 2011 to 2013. The genetic relatedness, serotype dominance and antibiotic resistance of isolates were investigated. A total of 46 strains were isolated from environmental samples (e.g., sediment, seawater and shrimp), whereas 249 strains were obtained from stools of patients with gastroenteritis. Nine different O serogroups and 16 serovars were identified. Serovars O3:K6 and O6:K46 were identified in both environmental and clinical strains. Whereas most environmental isolates carried the *tdh* gene (71.74%, 33/46), only three (6.52%) belonged to pandemic clones (O3:K6, O3:KUT and OUT:KUT). In contrast, 81.1% (202/249) of clinical isolates belonged to pandemic serotypes, with O3:K6 (*tdh*, *toxRS/new*, and/or *orf8*) representing the predominant serovar (97%, 196/202). This prevalence of pathogenic (*tdh* and/or *trh* positive) and O3:K6 pandemic *V. parahaemolyticus* isolates in this study were similar to those found from 2004 to 2010. As investigated by REP-PCR, genetic lineages of selected O3:K6 strains isolated in this study and some isolated earlier were nearly identical. Antimicrobial susceptibility testing showed that most strains (93.8%) were resistant to ampicillin but sensitive to *chloramphenicol* (98.8%). Multidrug resistance significantly increased from 8.6% (2004–2010) to 22.93% (2011–2013; *p* < 0.05). Our data indicate that pandemic O3:K6 clone has endemically established in the Pacific Coast of Mexico.

## Introduction

*Vibrio parahaemolyticus* is a Gram stain-negative bacterium autochthonous of marine and estuarine environments worldwide (Kaneko and Colwell, 1973, 1978; Joseph et al., 1982). While the majority of environmental strains are innocuous members of the marine microbiota, small subpopulations are opportunistic pathogens of humans (Johnson et al., 2008). Potentially virulent strains are commonly differentiated from likely avirulent strains by the presence of the thermostable direct (*tdh*) and/or *tdh*-related (*trh*) hemolysin genes (Shirai et al., 1990; Bej et al., 1999). Acute gastroenteritis is the most common manifestation of illness and often associated with the consumption of raw or undercooked oysters, which can bioaccumulate the bacterium through filter-feeding (Daniels et al., 2000; Su and Liu, 2007; Iwamoto et al., 2010).

Previously, *V. parahaemolyticus* infections have been typically sporadic cases attributed to multiple serotypes, with at least 13 O serogroups and 71 K serotypes detected (Ishibashi et al., 2000). There was not a clear association between *V. parahaemolyticus*-mediated infection and serovars until 1996. Serogroup O3:K6 was first isolated in 1996 from diarrhea patients in Kolkata, India (Okuda et al., 1997) and subsequently worldwide. Since then an increasing incidence of gastroenteritis caused by the serogroup O3:K6 has been reported in many countries, including Africa (Ansaruzzaman et al., 2005), Europe (Martinez-Urtaza et al., 2004, 2005), and Latin America (Gonzalez-Escalona et al., 2005). Serotype O3:K6 strain was then identified as a dominant pandemic clone from clinical cases of *V. parahaemolyticus*-induced diarrhea reported globally (Okuda et al., 1997; Chowdhury et al., 2000).

Up to now, a wide variety of O3:K6 clonal derivatives, including O4:K68, O1:K25, O1:K26, and O1:KUT, have been recognized as the predominant group responsible for most outbreaks since 1996 (Okuda et al., 1997; Matsumoto et al., 2000; Okura et al., 2004;Ansaruzzaman et al., 2005; Hayat Mahmud et al., 2006). Pandemic strains typically belong to serotype O3:K6 and encodes a unique *orf8* gene (Nasu et al., 2000). It has been hypothesized that *orf8* encodes for an adherence protein that increases the ability of *V. parahaemolyticus* to adhere to host intestinal cells or the surfaces of marine plankton (Nasu et al., 2000; Yeung et al., 2002). Several studies reported that the *tox*RS operon of pandemic strains contains a unique sequence, thereby referred as *tox*RS/new, encoding transmembrane proteins involved in the regulation of virulence associated genes (Chowdhury et al., 2000; Okura et al., 2003, 2005). In general, an isolate possessing both *tdh* and *toxRS*/new can be considered as a pandemic strain (Okura et al., 2003). Another known virulence gene, *trh*, is not specific to pandemic strains and it is rarely present in environmental strains compared to clinical ones (DePaola et al., 2000; Parvathi et al., 2006).

In Mexico, the first outbreak of gastroenteritis caused by the pandemic strain of *V. parahaemolyticus* O3:K6 was reported in 2004 (Cabanillas-Beltran et al., 2006). More than 1230 cases of infection with *V. parahaemolyticus* were associated to consumption of contaminated seafood in a relatively small geographic area in southern Sinaloa (Cabanillas-Beltran et al., 2006). The incidence of *V. parahaemolyticus* infections in Mexico was unknown until 2004 when the O3:K6 pandemic strain with the *tdh* virulence gene was detected in this region. In subsequent years, recurrent sporadic cases has been detected in both South and North areas with the pandemic strain O3:K6 causing >79% of reported cases between 2004 and 2010 (Velazquez-Roman et al., 2012).

In an effort to understand the prevalence and dissemination of *V. parahaemolyticus* strains (toxigenic and pandemic O3:K6) we have characterized since 2004 strains of *V. parahaemolyticus* isolated from both clinical cases and environmental samples obtained from South and North areas of the Sinaloa state (Velazquez-Roman et al., 2012). The present report describes a more extensive investigation that evaluated the prevalence of *V. parahaemolyticus* strains in clinical and environmental samples collected from 2011 through 2013 from along all Sinaloa state. Our studies characterized the isolates by serotyping, investigated their antimicrobial susceptibility or non-susceptibility and assessed the presence of toxigenic and pandemic genetic markers. We also investigated the genetic relationships of strains isolated between 2004 and 2010 to those investigated in this study and isolated in 2011–2013. Our results indicate the persistence in the environment and clinical cases of the O3:K6 pandemic strain in Northwest Mexico from 2004 to 2013 To our knowledge, this is the first report describing that the pandemic O3:K6 clone has endemically established in the Pacific Coast of Mexico and causes most *V. parahaemolyticus* attributable diarrhea cases.

## Materials and Methods

### Area of Study, Collection of Environmental Samples, and Stool Samples

**FIGURE 1:**
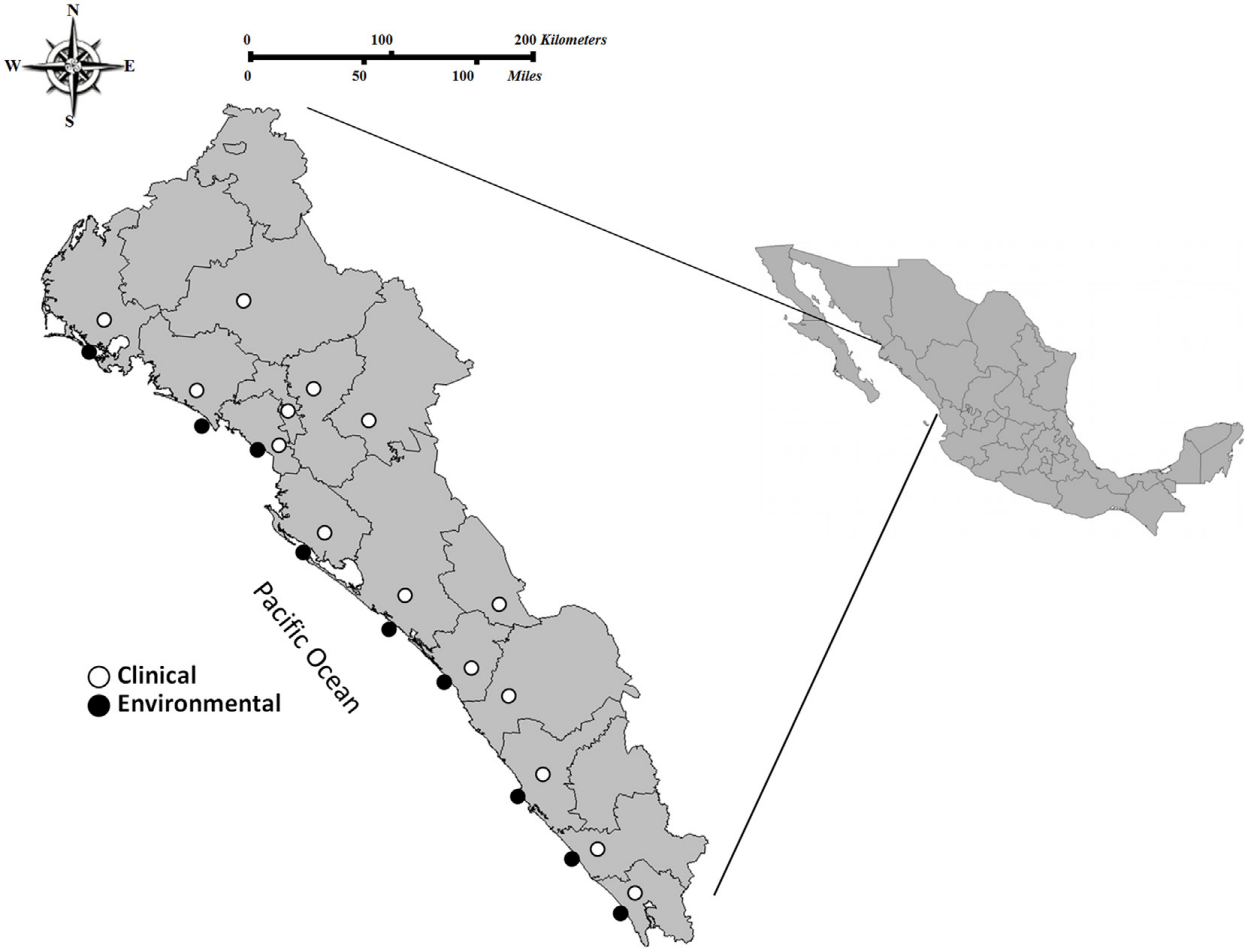
Area of study and locations of the sampling sites in the Pacific Northwest coast of Mexico. Clinical cases (stools specimens or rectal swabs; *n* = 10,521 of which 249 were positive isolates for *Vibrio parahaemolyticus*) and environmental samples (seawater, sediment, and shrimp; *n* = 1,895 of which 46 were positive isolates for *V. parahaemolyticus*) collected from 2011 through 2013 in the coastal regions of northern to southern Sinaloa.

This study was performed in the state of Sinaloa, which is located in Northwest Mexico. Sinaloa has over 650 km of coastline, with most of it (∼75%) facing the Sea of Cortez and the rest (∼25%) bordering the Pacific Ocean. Sample collection was performed in eleven sites from the southern to the northern region in Sinaloa, during the years of 2011, 2012, and 2013. Regions sampled include were leading shrimp producers in Sinaloa are located; clinical cases were also detected near these regions. A total of 1,895 environmental samples (shrimp *N* = 204, sediment *N* = 9, and seawater *N* = 1,682) were collected (**Figure 1**). Stool specimens or rectal swabs (*N* = 10,521) were collected in Cary-Blair transport medium from persons with clinical gastroenteritis who had eaten seafood and requested attention in public-sector health care agencies during the period 2011–2013 (**Figure 1**). Written informed consent was obtained from patients or their families. Procedures for collection of stool samples were approved by the ethics committee of the Faculty of Medicine-UAS and the Sinaloa State Public Health Laboratory.

### Bacteriological Analyses

Samples were processed following procedures found in the Bacteriological Analytical Manual of the Food and Drug Administration (Kaysner and De Paola, 2004) and as described by Canizalez-Roman et al. (2011). Briefly, 50 g of shrimp, or sediment samples, or 50 mL of seawater, were homogenized with 450 mL of sterile alkaline peptone water (APW; pH 8.6) in a Stomacher 400 circulator. The APW homogenate was incubated at 37°C for 6–8 h. Stool samples, or rectal swabs, were placed in Cary-Blair transport medium and transported at room temperature (RT) to the laboratory within 2 h and immediately processed. These specimens were also enriched in APW (pH 8.6) for 6–8 h at 37°C. After incubation, the enrichment broths were streaked onto thiosulfate-citrate-bile salts-sucrose (TCBS) agar plates and/or CHROMagar Vibrio (CV) medium (CHROMagar, Paris, France) and incubated at 37°C for 18–24 h. At least three typical colonies of *V. parahaemolyticus* were isolated from each plate and subjected to identification by biochemical test and polymerase chain reaction (PCR) as mentioned below. After identification of *V. parahaemolyticus* a single colony from each sample was used to continue the analysis (serotyping and virulence genes).

### Extraction and Purification of Chromosomal DNA

Chromosomal DNA was extracted using the Wizard genomic DNA purification kit (Promega Corp.) according to the manufacturer’s instructions. Briefly, strains were inoculated in LB broth containing 3% NaCl an incubated overnight at 37°C. This culture (3 mL) was pelleted by centrifugation at 16,000 × *g* for 5 min. Cells were lysed at 80°C in nucleic lysis solution. RNase solution was added to the cell lysate, followed by incubation at 37°C for 1 h and cooling at RT. Protein precipitation solution was added to the RNase-treated cell lysate and vortexed vigorously. DNA was precipitated by adding 0.6 volumes of isopropanol at RT and then washed with 70% ethanol; air dried, and solubilized using DNA rehydration solution. Our DNA preparations were stored at -20 or -80°C until use.

### PCR Assays

Polymerase chain reaction amplification was performed in 25 μL reactions consisting of 1X GoTaq green master mix (Promega), primers targeting either the *tl* gene (Bej et al., 1999), pR72H plasmid (Lee et al., 1995; Robert-Pillot et al., 2002), *tdh* or *trh* genes (Bej et al., 1999), and 0.5 μL of purified genomic DNA template, with the remaining volume consisting of molecular biology grade water. PCR was routinely carried out in a Thermal Cycler C1000 (Bio-Rad Laboratories, Hercules, CA, USA) under the following cycling conditions: an initial period of DNA denaturation at 94°C for 3 min, followed by 35 cycles of 1 min at 94°C, 1 min at 58°C, and 1 min at 72°C, and a final extension of 5 min at 72°C. PCR assays to amplify the *toxRS/new* and *orf8* genes (pandemic markers) were performed using specific primers previously reported to detect *toxRS/new* (Matsumoto et al., 2000) sequence unique to the pandemic clone of *V. parahaemolyticus* and the *orf8* (Myers et al., 2003) sequence of phage f237, respectively. PCR conditions for these assays were the following: for the *tox*RS/*new* gene, initial denaturation at 94°C for 3 min, followed by 25 cycles of 30 s at 94°C, 30 s at 45°C, and 1 min at 72°C, with a final extension of 5 min at 72°C, and for the *orf8* gene, denaturation at 94°C for 3 min, followed by 30 cycles of 30 s at 94°C, 30 s at 55°C, and 30 s at 72°C, with a final extension of 5 min at 72°C (Matsumoto et al., 2000; Myers et al., 2003). Ten microliter aliquots of each amplification product were separated by electrophoresis in 2% agarose gels. Ethidium bromide staining (0.5 mg/mL) allowed for the visualization of DNA fragments with a digital imaging system (Gel Doc EZ imager, Bio-Rad, Hercules, CA, USA). The sizes of the PCR fragments were compared against a 50-bp DNA ladder (Promega DNA step ladder).

### Serotyping

Serotyping of *V. parahaemolyticus* isolates was done by using a commercially available *V. parahaemolyticus* antiserum test kit (Denka Seiken, Tokyo, Japan) with O1–O11 antisera and 71 K antisera according to the manufacturer’s instructions. Briefly, strains were grown overnight at 37°C on LB agar containing 3% NaCl. A pool of colonies was suspended in 1 mL of saline and then split in two 500 μL aliquots. For serotyping, an aliquot was heated up to 121°C for 1 h for O serotyping; if the serotype could not be obtained, the bacterial lysate was heated for an additional hour and then used for O serotyping. The second aliquot was used for serotyping based on the K antigen.

### REP-PCR

More than 50% (*n* = 150) of strains isolated between 2004–2010 and 2011–2013 were selected for repetitive extragenic palindromic PCR (REP-PCR) analysis. Of these only nine strains are shown in the results section. Strains from the 2004 to 2010 period were obtained from a previous study (Velazquez-Roman et al., 2012) and from the MEMC (Medical and Enviromental Microorganism Collection, School of Medicine, Autonomous University of Sinaloa, Culiacan, Sinaloa, México). Seven strains belonged to pandemic O3:K6 strains, one strain sharing serogroup and pandemic characteristics O3:KUT isolated from shrimp sample, and a clinical isolate belonging to serotype O1:K20. Reactions were performed with primers REP-1D, 5-NNN RCG YCG NCA TCM GGC-3, and REP-2D, 5-RCG YCT TAT CMG GCC TAC-3, where M is A or C, R is A or G, Y is C or T, and N is any nucleotide (Wong and Lin, 2001; Maluping et al., 2005). PCR was routinely carried out in a Thermal Cycler C1000 (Bio-Rad Laboratories, Hercules, CA, USA) under the following cycling conditions: an initial period of DNA denaturation at 95°C for 7 min, followed by 35 cycles of 0.30 min at 94°C, 1 min at 45°C, and 8 min at 72°C, and a final extension of 10 min at 72°C (Wong and Lin, 2001; Maluping et al., 2005). PCR products were resolved by gel electrophoresis (1.5% agarose) buffered in Tris acetate EDTA (TAE) at 80 V for 2 h, stained with ethidium bromide (0.5 mg/mL) allowed for the visualization of DNA fragments with a digital imaging system (Bio-Rad Gel Doc EZ Imager, Wayne, PA, USA).

### Antibiotic Susceptibility Testing

To evaluate antimicrobial-susceptibility of *V. parahaemolyticus* strains, 65 clinical and 87 enviromental isolates from 2004 to 2010 (from MEMC, a previous study (Velazquez-Roman et al., 2012), and 77 clinical and 32 enviromental isolates from 2011 to 2013, were tested by a standard disk diffusion method on Mueller-Hinton II agar (CLSI, 2011). The antibiotic sensi-disk (BD BBL, Sensi-Disc, Becton, Dickinson and Company, USA) used were the following: ampicillin (10 μg), tetracycline (30 μg), trimethoprim–sulfamethoxazole (1.25 μg/23.75 μg), chloramphenicol (30 μg), nalidixic acid (30 μg), ciprofloxacin (5 μg), ceftazidime (30 μg), gentamicin (10 μg), and cefotaxime (30 μg). In the absence of Clinical and Laboratory Standards Institute (CLSI) definitive standards for interpreting *V. parahaemolyticus* susceptibility to antibiotics, zone diameters were determined and recorded as sensitive, intermediate, or resistant according to established standards for *V. cholerae* and *Enterobacteriaceae*. The following *V. parahaemolyticus* strains were used as a control organism: ATCC 17802, (*tdh*^-^) and multidrug resistant strain 727 (Leon-Sicairos et al., 2009).

### Statistical Analysis

All statistical analysis was performed using SPSS v.20.0 (IBM Corp., Armonk, NY, USA). We carried out Chi-square to evaluate significance.

## Results

### Isolation of *V. parahaemolyticus* from Environmental and Stool Samples

From 2011 to 2013, a total of 1,895 environmental samples were analyzed for the presence of *V. parahaemolyticus* strains; these samples included 204 shrimp, 1682 seawater and nine sediment samples (**Figure 1**). Overall, *V. parahaemolyticus* strains were isolated from 2.4% (*N* = 46) of samples. Of these 46 strains, 38 (82.6%) were obtained from shrimp samples, 5 (10.9%) from sediment, and 3 (6.5%) from seawater. In clinical samples taken during the same period, *V. parahaemolyticus* strains were isolated in 249 (2.4%) out of 10,521 stool specimens or rectal swabs collected from persons with gastroenteritis who had eaten seafood. The presence of *V. parahaemolyticus* in both, environmental samples and in cases of diarrhea by this bacterium were detected from southern to northern Sinaloa state (**Figure 1**).

### Virulence Genes, Serotypes and Pandemic Characteristics of *V. parahaemolyticus* Isolates

Based on the presence or absence of virulence genes, we classified the isolates into three groups: pandemic (*tdh*^+^, *toxRS/new*^+^ and/or *orf8*^+^), pathogenic (*tdh*^+^ and/or *trh*^+^), and non-pathogenic strains (*tdh*^-^ and *trh*^-^). Among environmental *V. parahaemolyticus* strains, three strains (6.5%) were identified as pandemic isolates. One of these strains belonged to serotype O3:K6 and carried the *tdh*, t*oxRS/new*, and *orf8* genes (isolated from shrimp), whereas two pandemic O3:KUT strains carried the *tdh*, *toxRS/new* and/or *orf8* genes (isolated from sediment and shrimp). A total of 65.2% (30/46) of environmental isolates carried the virulence *tdh* gene and therefore are considered as pathogenic strains. We did not detect isolates encoding the *trh* gene (**Table 1**). Approximately 28% (13/46) of environmental isolates were non-pathogenic. The most prevalent serovars were O3:KUT (23.9%, 11/46) and O5:K30 (10.8%, 5/46). Non-typeable strains represented the 39.13% (18/46; **Table 1**). Regarding *V. parahaemolyticus* strains isolated from diarrhea cases, 81.1% (202/249) of these isolates were identified as pandemic serotypes (**Table 1**). Of these, 97% (196/202) belonged to serovar O3:K6, carrying the *tdh*, *toxRS/*new and/or *orf8* genes. One isolate belonged to serovar O3:K29 (*tdh^+^* and *toxRS/new^+^*), and one isolate belonged to serotype OUT:KUT (*tdh^+^*, *toxRS/new^+^*, and *orf8^+^*). A total of 16.1% (40/249) of these clinical isolates were pathogenic strains (*tdh^+^* and/or *trh^+^*) including several serotypes (e.g., O1:KUT, O4:K12, O4:K29, O4:K55, O6:K18, O10:KUT, OUT:KUT, OUT:K53, O1:K56, O3:KUT, O4:KUT, O8:K21). Only few clinical isolates, 2.8% (7/249), were classified within the non-pathogenic group. Unlike serovars detected in environmental isolates, pandemic serotype O3:K6 (80.3%, 200/249) was the most prevalent among those isolated from clinical samples (**Table 1**). Serotypes O1:KUT, O2:KUT, O3:KUT, O3:K6, O6:K46, and OUT:KUT were isolated from both environmental samples and stool samples.

**Table 1:** Serovar and virulence attributes of 295 *Vibrio parahaemolyticus* strains isolated between 2011 and 2013.

O serogroup and serovar	Total no. of isolates	Presence of each virulence gene				No. of clinical isolates (from feces)	No. of environmental isolated from:		


		*tdh*	*trh*	*toxRS/new*	*Orf8*		Sh	Sw	S
**O1**
O1:KUT	2	+	--	-	-	1	1		
	1	-	-	-	-		1		
	1	+	+	-	-	1			
O1:K20	1	-	-	-	-	1			
O1:K56	1	+	+	-	-	1			
**O2**
O2:KUT	2	+	-	-	-		2		
	1	-	-	-	-	1			
**O3**
O3:KUT	2	-	-	-	-	1	1		
	6	+	-	-	+		5		1
	2	+	-	-	-			1	1
	1	+	-	+	-				1
	1	+	-	+	+		1		
	1	+	+	-	-	1			
O3:K6	192	+	-	+	+	191	1		
	5	+	-	+	-	5			
	21	+	-	-	+	21			
	4	+	+	+	+	4			
O3:K29	1	+	-	+	-	1			
O3:K30	1	-	-	-	-	1			
O3:K33	1	+	-	-	+		1		
	2	-	-	-	-		2		
O3:K54	1	-	-	-	-	1			
**O4**
O4:KUT	2	+	+	-	-	2			
O4:K4	1	+	-	-	-				1
O4:K12	1	+	-	-	-	1			
O4:K29	1	+	-	-	-	1			
O4:K55	1	+	-	-	-	1			
O4:K63	1	-	-	-	-	1			
**O5**
O5:K30	2	-	-	-	-		2		
	1	+	-	-	+		1		
	2	+	-	-	-		2		
**O6**
O6:K18	1	+	+	-	-	1			
	1	+	-	-	-	1			
O6:K46	2	-	-	-	-	1	1		
**O8**
O8:K21	1	+	+	-	-	1			
**O10**
O10:KUT	2	+	-	-	-	2			
**O11**
O11:KUT	1	-	-	-	-		1		
**OUT**
OUT:KUT	14	+	-	-	-	2	11	1	
	1	-	+	-	-	1			
	1	+	-	+	+	1			
	1	+	-	-	+		1		
	1	+	+	-	-	1			
	5	-	-	-	-		3	1	1
OUT:K6	1	+	-	-	-		1		
OUT:K53	1	+	-	-	-	1			
							38	3	5

**TOTAL**	**295**					**Total clinical: 249**	**Total environmental: 46**

*Sh, Shrimp; Sw, Seawater; S, Sediment*.

### Prevalence of O3:K6 Pandemic Clone and Pathogenic Strains between 2004–2010 and 2011–2013

**FIGURE 2:**
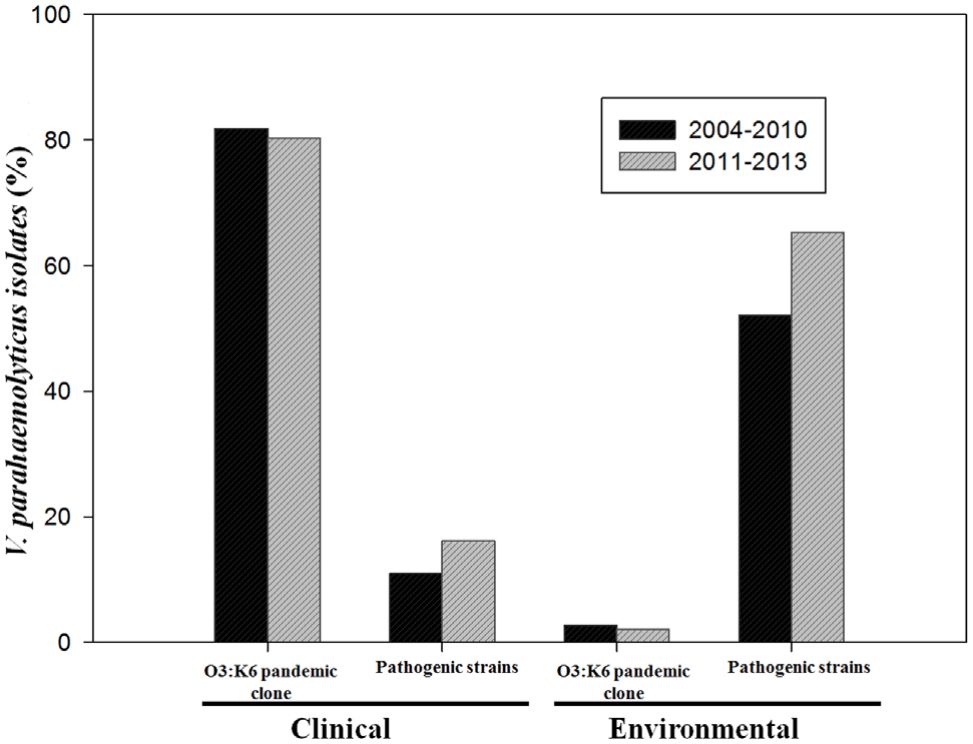
Prevalence of pandemic *Vibrio parahaemolyticus* O3:K6 and pathogenic strains isolated from environmental and clinical samples that were collected in periods 2004–2010 or 2011–2013.

The pandemic clone O3:K6 serotype was the most prevalent strain isolated from gastroenteritis cases in both periods 2004–2010 (81.8%) and 2011–2013 (80.3%). Among environmental strains the prevalence of serotype O3:K6 was also similar, 2.7 or 2.1%, for those isolated in 2004–2010, or 2011–2013, respectively (**Figure 2**). The percentage of clinical pathogenic strains isolated during the period 2011–2013 (16.1%) was slightly higher than that obtained during the period 2004–2010 (11%) but statistical analysis revealed no significant difference (*p* > 0.05). In the case of environmental pathogenic strains the prevalence increased from 52% in 2004–2010 to 65.3% in our period of analysis 2011–2013 (**Figure 2**). Similarly, no statistically significant difference was detected (*p* > 0.05). This indicates that the incidence of *V. parahaemolyticus* infection by the pandemic strains (O3:K6) in this region of Mexico had remained constant since 2004. It is noteworthy that between 2004 and 2013, O3:K6 strains were isolated from clinical samples in high proportions (80.3–81.8%) whereas pathogenic strains were detected in low proportions (11–16.1%). Conversely, in environmental samples the pandemic clone O3:K6 was detected in low proportions (2.1–2.7%) and pathogenic strains were detected in high proportions (52–65.3%).

### REP-PCR Typing of Clinical and Environmental *V. parahaemolyticus* Strains

**FIGURE 3:**
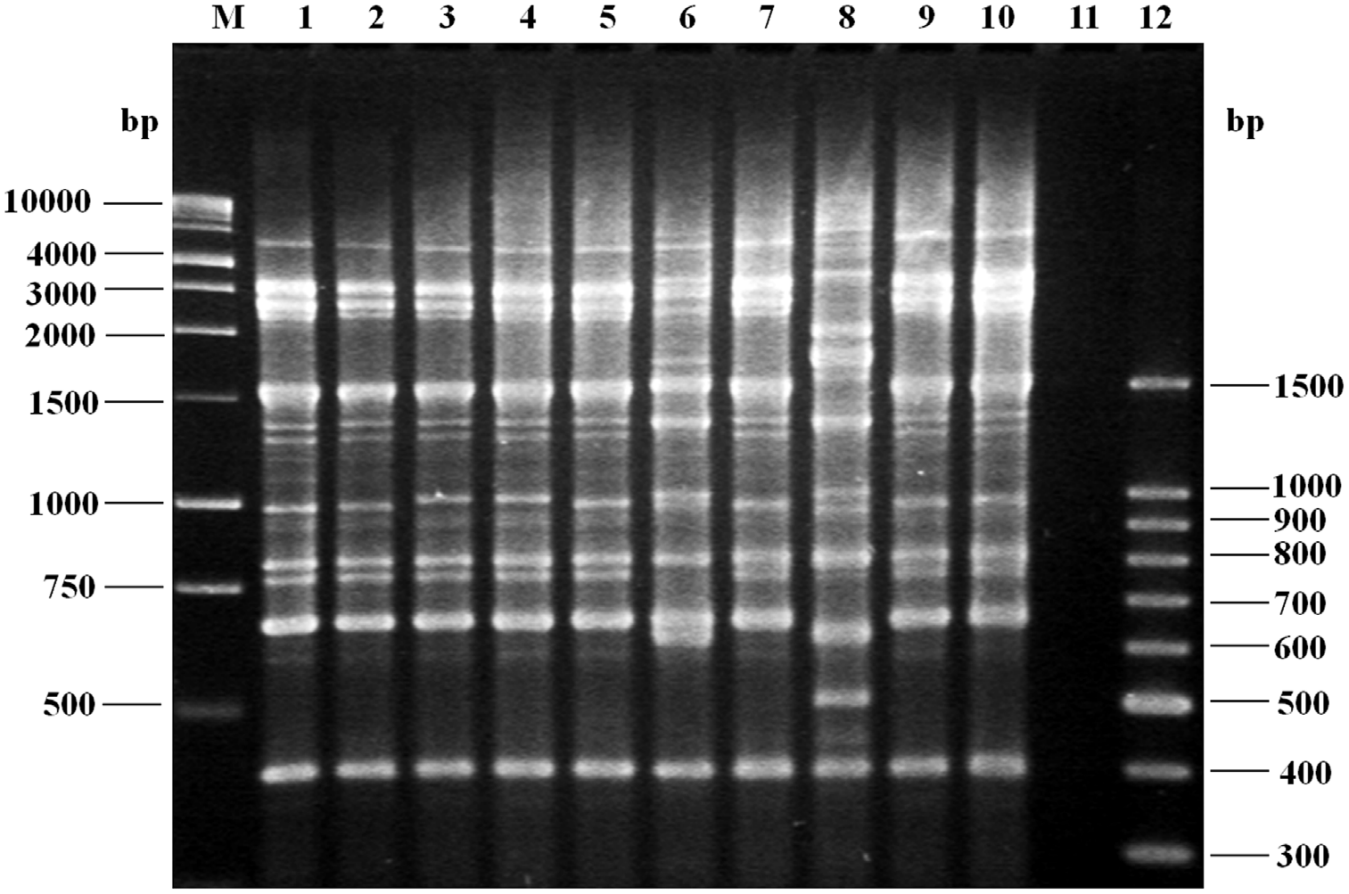
Agarose gel (1.5%) electrophoresis showing the results of polymerase chain reaction (PCR) amplification of representative fingerprint patterns for the REP-PCR of *Vibrio parahaemolyticus* strains of serotype O3:K6 (*tdh^+^*, *toxRS/new^+^*, *orf8^+^*, and *trh^-^*), isolated from 2004 to 2013. Lane M, 1 kb molecular size markers; lane 1, clinical isolate (2004, O3:K6); lane 2, clinical isolate (2006, O3:K6); lane 3, clinical isolate (2008, O3:K6); lane 4, clinical isolate (2010, O3:K6); lane 5, clinical isolate (2011, O3:K6); lane 6, shrimp isolate (2011, O3:KUT, *tdh^+^*, *toxRS/new^+^*, *orf8^+^*, and *trh^-^*); lane 7, clinical isolate (2012, O3:K6); lane 8, clinical isolate (2012, O1:K20, *tdh^-^*, *toxRS/new^-^*, *orf8^-^*, and *trh^-^*); lane 9, clinical isolate (2013, O3:K6); lane 10, control strain *V. parahaemolyticus* RIMD 2210633 (*tdh^+^*, *toxRS/new^+^*, *orf8^+^*, and *trh^-^*); lane 11, negative control (no DNA template added to the rep-PCR reaction); lane 12, molecular size markers.

To investigate whether infections due to pandemic isolates were caused by genetic related clones throughout the years, DNA fingerprints of 150 strains obtained during 2004–2013 were examined using REP-PCR, but only seven randomly selected O3:K6, one O3:KUT and one O1:K20 isolates are shown in the **Figure 3**. Our REP-PCR studies revealed 11 discernible products (i.e., PCR bands) ranging in size from 400 to 3,000 bp. Several REP-PCR products with molecular size of 600, 750, and 1500 bp were common to most strains, while products of 400, 800, 1000, and 3000 bp were present in all *V. parahaemolyticus* strains (**Figure 3**). One REP-PCR banding pattern was obtained for O3:K6 (*tdh^+^*, *toxRS/new^+^*, *orf8^+^*, and *trh^-^*) strains; these seven isolates (**Figure 3**, lanes 1–5, 7, 9), yielded an identical banding pattern to that observed for the control strains (**Figure 3**, lane 10). A second banding pattern comprised one isolate, O3:KUT (*tdh^+^*, *toxRS/new^+^*, *orf8^+^* and *trh^-^*), (**Figure 3**, lane 6) and a third REP-PCR banding pattern was obtained with one isolate, O1:K20 (*tdh*^-^, *toxRS/new*^-^, *orf8*^-^, and *trh*^-^; **Figure 3**, lane 8). Similar REP-PCR banding pattern were observed when REP-PCR was repeatedly performed, at least three times, demonstrating the reproducibility of our data. Except for the O3:KUT, lane 6 and O1:K20, lane 8 which displayed non-identical REP-PCR profiles, isolates from any year with the same serotype mostly produced identical REP-PCR profiles.

### Antibiotic Resistance Profiles of *V. parahaemolyticus* Strains

Of the environmental and clinical strains tested, a significant increase in cefotaxime resistance was observed from 2004–2010 to 2011–2013 (*p* < 0.05) and most isolates were resistant to ampicillin (**Table 2**). However, among clinical strains a significantly ampicillin-resistance decreased was observed from 2004–2010 to 2011–2013 (*p* < 0.05). Low resistance was determined for gentamicin, nalidixic acid, sulfamethoxazole-trimethoprim, ceftazidime, chloramphenicol, and tetracycline from 2004–2010 to 2011–2013 (**Table 2**). Clinical and environmental isolates were all susceptible to ciprofloxacin or chloramphenicol, respectively.

**Table 2:** Distribution of antibiotic resistance among clinical and environmental *Vibrio parahaemolyticus* strains isolated from 2004–2010 and 2011–2013 periods.

Antimicrobial agents	Sample type			
	Environmental		Clinical	
	2004–2010 *n* = 87 (%)	2011–2013 *n* = 32 (%)	2004–2010 *n* = 65 (%)	2011–2013 *n* = 77 (%)
**Aminoglycoside**
*Gentamicin*	2 (2.3%)	1 (3.1%)	0	3 (3.9%)
**Quinolones and fluoroquinolones**					
*Ciprofloxacin*	1 (1.1%)	0	0	0
*Nalidixic acid*	1 (1.1%)	0	1 (1.5%)	4 (5.2%)
**Sulfonamides and potentiated sulfonamides**
*Sulfamethoxazole-trimethoprim*	2 (2.3%)	0	0	2 (2.6%)
**Tetracyclines**					
*Tetracycline*	1 (1.1%)	0	1 (1.5%)	1 (1.3%)
**Beta lactams**					
*Ampicillin*	78 (89.7%)	32 (100%)	65 (100%)∗	70 (90.1%)∗
**Cephalosporins**
*Ceftazidime*	3 (3.4%)	0	0	4 (5.2%)
*Cefotaxime*	3 (3.4%)∗	7 (21.9%)∗	4 (6.1%)∗	14 (18.2%)∗
**Phenicols**
*Chloramphenicol*	0	0	0	3 (3.9%)

*^∗^Significant at 0.05 level*.

Regarding overall antibiotic resistance, most environmental (>78%) and clinical strains (>70%) were non-susceptible to at least one antibiotic (**Table 3**). This high prevalence of resistance decreased in strains isolated in 2011–2013 in comparison to previous period, 2004–2010, with an expected increase on the prevalence of strains resistant to two or more antibiotics (multidrug-resistant; **Table 3**). The increased prevalence, however, of strains with resistance to two antibiotics was only statistically significant among clinical isolates (*p* < 0.05). Importantly, strains with resistance to 5 or 7 antibiotics were detected among clinical strains isolated in the period 2011–2013 (**Table 3**).

**Table 3:** Drug resistance and multidrug-resistance among clinical and environmental *Vibrio parahaemolyticus* strains isolated from 2004–2010 and 2011–2013 periods.

Number of drugs resistant to:	Sample type			
	Environmental		Clinical	
	2004–2010*n* = 87 (%)	2011–2013*n* = 32 (%)	2004–2010*n* = 65 (%)	2011–2013*n* = 77 (%)
0	7 (8.1%)	0	0	5 (6.5%)*
1	72 (82.8%)	25 (78.1%)	60 (92.3%)*	54 (70.1%)*
2	6 (6.9%)	6 (18.8%)	4 (6.2%)*	13 (16.9%)*
3	1 (1.1%)	1 (3.1%)	1 (1.5%)	3 (3.9%)
4	1 (1.1%)	0	0	0
5	0	0	0	1 (1.3%)
6	0	0	0	0
7	0	0	0	1 (1.3%)
≥8	0	0	0	0

*^∗^Significant at 0.05 level*.

## Discussion

In Mexico, the first outbreak of gastroenteritis caused by pandemic *V. parahaemolyticus* strain O3:K6 was reported in a relatively small geographical area of the Southern part of the Sinaloa State (Velazquez-Roman et al., 2012). Since its arrival back in 2004, Sinaloa has experienced recurrent sporadic cases of gastroenteritis caused by *V. parahaemolyticus* strains which have gradually spread from south to north from 2004 to 2010 (Velazquez-Roman et al., 2012). The present study conducted an epidemiological surveillance of *V. parahaemolyticus* strains in both environmental and clinical samples along the Pacific coast of Sinaloa from 2011 to 2013. We demonstrate that the pandemic clone O3:K6 (encoding the *tdh* and *tox*RS/*new* genes and with or without *orf8*) still remains the most prevalent serotype isolated from cases of *V. parahaemolyticus*-induced diarrhea cases. The pandemic clone has endemically established in the Pacific Coast of Mexico. Furthermore, most strains were resistant to ampicillin and resistance to multiple first-line antibiotics significantly increased from 2004–2010 to 2011–2013. These observations represent, to the best of our knowledge, the first report demonstrating 10 years of persistence of the pandemic clone O3:K6 in the Mexico’s pacific coast.

As in our previous study where most strains isolated from 2004 to 2010 belonged to the O3:K6 serotypes, most strains isolated from 2011 to 2013 were serotype O3:K6 (Velazquez-Roman et al., 2012). The prevalence of O3:K6 pandemic and pathogenic strains isolated from environmental and clinical samples was not significantly different from that detected in 2004–2010. This indicates that (1) the incidence of *V. parahaemolyticus* infection by the pandemic strains remains similar and (2) that the pandemic clone is a permanent resident of the environment in this region of Mexico. We hypothesize that the presence of pandemic strains in the environment is at least partially due to shedding in the feces of patients with gastroenteritis.

Regional persistence of O3:K6 pandemic strains have been reported in different geographic areas. For example, O3:K6 was the predominant serovar in studies conducted in Peru in 2007 (Gil et al., 2007), China (2007–2012; Li et al., 2014), and other Asian countries (Arakawa et al., 1999; Chowdhury et al., 2000; Wong et al., 2000) as well as in Chile when investigated from 2004 to 2009 (Gonzalez-Escalona et al., 2005; Cabello et al., 2007; Fuenzalida et al., 2007; Garcia et al., 2009). A follow up study in Chile conducted in by Harth et al. (2009) made an interesting observation of serotype replacement. The authors reported a decrease in outbreaks caused by O3:K6 but an increase of cases caused by pandemic isolates belonging to serotype O3:K59. In contrast, no change in the prevalence of the O3:K6 pandemic clone was observed in Northwest Mexico from 2004 to 2013. Our studies also identified new serovariants (O3:K29 and OUT:KUT) emerging with virulence attributes (*tdh* positive, *tox*RS/*new* positive and/or *orf8* positive) of pandemic strains. The emergence of new serovariants warrants further investigation since clones can potentially produce outbreaks along the northern Mexican coastline of the Pacific Ocean and spread to South Mexico or head North to US and Canada.

Results showed that the serovars of *V. parahaemolyticus* in environmental and clinical isolates were abundant and variable. We identified 10 novel serovars (from 2011 to 2013) in the area that were not isolated in our previous investigation conducted during 2004–2010. These newly identified serovars were isolated from diarrhea cases, *N* = 7, (O1:K20, O3:K30, O3:K54, O4:K29, O4:K55, O6:K18, and OUT:K53) and three from environmental samples (O4:K4, O5:K30, and OUT:K6). Data from the present study are in accordance with other reports in which *V. parahaemolyticus* environmental strains show a high serological variability (Nair et al., 2007; Chao et al., 2009; Garcia et al., 2009).

Previous studies have demonstrated that up to 90% of clinical strains encode the *tdh* and/or *trh* gene (Okuda et al., 1997; Chao et al., 2009; Garcia et al., 2009; Velazquez-Roman et al., 2012; Li et al., 2014; Pazhani et al., 2014), whereas their presence in environmental isolates is rare (Shirai et al., 1990; DePaola et al., 2000; Yeung and Boor, 2004; Nair et al., 2007; Chao et al., 2009; Velazquez-Roman et al., 2012). More recently, however, an increased proportions (48–52%) of strains encoding virulence genes (i.e., *tdh* and/or *trh*) have been detected in environmental isolates obtained in Mexico and the US. (Paranjpye et al., 2012; Velazquez-Roman et al., 2012; Gutierrez West et al., 2013). Accordingly, our studies detected high prevalence of the *tdh* gene (encoding for the TDH hemolysin) as it was carried by 58.6% of all environmental strains isolated from 2004 to 2013. Besides this demonstrated high serodiversity in the environment of *V. parahaemolyticus* strains with pathogenic potential (i.e., non-O3:K6 strains encoding the *tdh* gene), the pandemic strain O3:K6 caused >81% of reported cases of gastroenteritis, attributable to *V. parahaemolyticus* between 2004 and 2013 in the Pacific Northwest coast of Mexico. The detection of *tdh* gene in environmental isolates suggests that *tdh* alone is not an adequate marker for potentially virulent *V. parahaemolyticus* strains (Paranjpye et al., 2012).

As expected, our studies found a high serodiversity of *V. parahaemolyticus* in the environment, including isolates obtained from shrimp, sediment, and seawater. It is worth to mention that in 2013 we observed a high mortality of cultured *Penaeus vannamei* in shrimp farms located in northern Mexico including the states of Nayarit, Sinaloa and Sonora. Mortality was due to acute hepatopancreatic necrosis disease (AHPND), which has also been referred to as early mortality syndrome (EMS), and the pathogen associated with EMS was *V. parahaemolyticus* (Gomez-Gil et al., 2014; Gomez-Jimenez et al., 2014; Nunan et al., 2014). Additional studies should provide a link between pathogenic traits of *V. parahaemolyticus* strains and/or serotype or serovars, if any, associated to this syndrome in shrimps.

Rep-PCR genomic fingerprinting is known to have a greater resolving power than serotyping (Maluping et al., 2005). Our rep-PCR studies intended to demonstrate genetic similarities, or differences, between pandemic strains isolated from the environment with those isolated from human cases of gastroenteritis. Whereas molecular divergence was noticed on the banding profile obtained from O1:K20 and O3:KUT strains, we obtained a similar rep-PCR profile in all O3:K6 pandemic isolates utilized which indicates that O3:K6 strains circulating in the environment have the same clonal origin than those infected patients and therefore a source of infection and transmission.

Another important contribution in our work was the investigation of susceptibility, or not, of the isolated *V. parahaemolyticus* strains to first-line antibiotics utilized in the region. In agree with our genetic evidences (i.e., rep-PCR) indicating genetic relationships of the isolated strains, our results revealed similar resistance patterns in both clinical and environmental isolates. Most *V. parahaemolyticus* isolates were resistant to ampicillin which was not a surprise as non-susceptibility to ampicillin is very common in *V. parahaemolyticus* strains isolated from enviromental and clinical samples (Okuda et al., 1997; Wong et al., 2000; Roque et al., 2001; Sun et al., 2013), suggesting that these drugs have a negligible role in the treatment of *V. parahaemolyticus*. In contrast, most isolates were sensitive to tetracycline, trimethoprim–sulfamethoxazole, chloramphenicol, nalidixic acid, ciprofloxacin, ceftazidime and gentamicin, which can be used as an alternative antibiotic therapy. Resistance to cefotaxime increased from 4.6% in 2004–2010 to 19.3% in 2011–2013. A similar prevalence of resistance to cefotaxime (20%) has also been reported in strains isolated in Italy from shellfish and clinical samples (Ottaviani et al., 2013). While the percentage of isolates expressing resistance to the newer generation of cephalosporins was relatively low, these antibiotics are considered to be some of the best defenses against the severe infections that these organisms can elicit, so even a small percentage of resistant isolates could be cause for concern. Therefore, all isolates must be tested for antimicrobial susceptibility to monitor resistance patterns of each antibiotic. In Mexico and others countries, patients suffering *V. parahaemolyticus* disease are treated with empiric antibiotic therapy which generates more resistance to first line antibiotics. Unlike other bacterial infections, little to nothing is reported about antibiotic resistance of *V. parahaemolyticus* in Mexico, and perhaps other Latin American countries, as clinical laboratories do not routinely test susceptibility to different classes of antimicrobial agents. Furthermore, until 2004 where we reported the first outbreak of gastroenteritis caused by *V. parahaemolyticus* in Mexico, there had not been other outbreaks published in the scientific literature (Velazquez-Roman et al., 2012, 2014). Prior to our studies, only few environmental strains had been isolated from water and fish in Mexico (Cabrera-Garcia et al., 2004).

To the best of our knowledge, our findings represent the first investigation in Mexico about the prevalence, pathogenic potential, and antimicrobial susceptibility over a 10-years period of continue surveillance of *V. parahaemolyticus* (pathogenic and pandemic O3:K6 clone) in both clinical specimens and environmental samples. Most gastroenteritis cases attributable to *V. parahaemolyticus* strains are caused by the same pandemic clone which warrants extended surveillance in the region and across the country. Continued monitoring of *V. parahaemolyticus* strains and their susceptibility to antibiotics seem to be necessary to unsure the best treatment, and prognosis, to patients with *V. parahaemolyticus* diseases in the area. This information should also be relevant to health authorities in the case of a local or multistate foodborne outbreak of *V. parahaemolyticus* gastroenteritis.
